# Combination therapy as a potential risk factor for the development of type 2 diabetes in patients with schizophrenia: the GOMAP study

**DOI:** 10.1186/s12888-018-1826-4

**Published:** 2018-08-02

**Authors:** Vasiliki Mamakou, Sophie Hackinger, Eleni Zengini, Evgenia Tsompanaki, Eirini Marouli, Ioannis Serafetinidis, Bram Prins, Athina Karabela, Eirini Glezou, Lorraine Southam, Nigel W. Rayner, Karoline Kuchenbaecker, Klea Lamnissou, Vassilis Kontaxakis, George Dedoussis, Fragiskos Gonidakis, Anastasia Thanopoulou, Nikolaos Tentolouris, Eleftheria Zeggini

**Affiliations:** 1 Medical School, National and Kapodistrian University Athens, 75 M. Assias Street, 115 27 Athens, Greece; 2 Dromokaiteio Psychiatric Hospital, 124 61 Athens, Greece; 3 Wellcome Sanger Institute, Hinxton, Cambridge, HH CB10 1 UK; 4 Department of Oncology and Metabolism, University of Sheffield, Sheffield, UK; 5 School of Information Sciences and Technology, Department of Statistics, Athens University of Economics and Business, 10434 Athens, Greece; 6 William Harvey Research Institute, Barts and The London School of Medicine and Dentistry, Queen Mary University of London, EC1M 6BQ, London, UK; 7 Department of Gastroenterology, Gennimatas General Hospital, 11527 Athens, Greece; 8 Dafni Psychiatric Hospital, 12462 Athens, Greece; 9 Wellcome Centre for Human Genetics, University of Oxford, Oxford, UK; 10 Oxford Centre for Diabetes Endocrinology and Metabolism, Oxford, UK; 11 National and Kapodistrian University of Athens, Department of Biology, Athens, Panepistimioupolis, AnoIlisia, 15771 Athens, Greece; 12 Early Psychosis Unit, 1st Department of Psychiatry, Eginition Hospital, Medical School, National and Kapodistrian University of Athens, 11527 Athens, Greece; 13 Department of Nutrition-Dietetics, Harokopio University, 17671 Athens, Greece; 14 1st Psychiatric Department, Eginition Hospital, Medical School, National and Kapodistrian University of Athens, 11527 Athens, Greece; 15 Diabetes Centre, 2nd Department of Internal Medicine, Hippokration General Hospital, Medical School, National and Kapodistrian University of Athens, 11527 Athens, Greece; 16 First Department of Propaedeutic Internal Medicine, National and Kapodistrian University of Athens, Medical School, Laiko General Hospital, 11527 Athens, Greece

**Keywords:** Type 2 diabetes, Schizophrenia, First generation antipsychotics, Second generation antipsychotics, Antidepressants, Mood stabilizers

## Abstract

**Background:**

Schizophrenia (SCZ) is associated with increased risk of type 2 diabetes (T2D). The potential diabetogenic effect of concomitant application of psychotropic treatment classes in patients with SCZ has not yet been evaluated. The overarching goal of the Genetic Overlap between Metabolic and Psychiatric disease (GOMAP) study is to assess the effect of pharmacological, anthropometric, lifestyle and clinical measurements, helping elucidate the mechanisms underlying the aetiology of T2D.

**Methods:**

The GOMAP case-control study (Genetic Overlap between Metabolic and Psychiatric disease) includes hospitalized patients with SCZ, some of whom have T2D. We enrolled 1653 patients with SCZ; 611 with T2D and 1042 patients without T2D. This is the first study of SCZ and T2D comorbidity at this scale in the Greek population. We retrieved detailed information on first- and second-generation antipsychotics (FGA, SGA), antidepressants and mood stabilizers, applied as monotherapy, 2-drug combination, or as 3- or more drug combination. We assessed the effects of psychotropic medication, body mass index, duration of schizophrenia, number of hospitalizations and physical activity on risk of T2D. Using logistic regression, we calculated crude and adjusted odds ratios (OR) to identify associations between demographic factors and the psychiatric medications.

**Results:**

Patients with SCZ on a combination of at least three different classes of psychiatric drugs had a higher risk of T2D [OR 1.81 (95% CI 1.22–2.69); *p* = 0.003] compared to FGA alone therapy, after adjustment for age, BMI, sex, duration of SCZ and number of hospitalizations. We did not find evidence for an association of SGA use or the combination of drugs belonging to two different classes of psychiatric medications with increased risk of T2D [1.27 (0.84–1.93), *p* = 0.259 and 0.98 (0.71–1.35), *p* = 0.885, respectively] compared to FGA use.

**Conclusions:**

We find an increased risk of T2D in patients with SCZ who take a combination of at least three different psychotropic medication classes compared to patients whose medication consists only of one or two classes of drugs.

**Electronic supplementary material:**

The online version of this article (10.1186/s12888-018-1826-4) contains supplementary material, which is available to authorized users.

## Background

Schizophrenia (SCZ) is associated with increased risk of obesity, impaired glucose tolerance, and type 2 diabetes (T2D) [1]. The prevalence of T2D among patients with SCZ in European populations is estimated to be up to 22.0% [2–6]. No information is available on T2D prevalence among psychiatric disease patients in Greece, whereas the current prevalence of T2D in the general Greek population has been estimated to be 11.97% (men 13.98%, women 9.25%), clearly indicating a major public health problem [7]. T2D is a major risk factor for cardiovascular disease and the fourth highest cause of excess mortality in patients with SCZ [8], leading to significantly shorter life expectancy [9]. Antipsychotic-naïve individuals with a first-episode SCZ often present with elevated fasting plasma glucose, impaired oral glucose tolerance and fasting plasma insulin levels, as well as insulin resistance, possibly owing to shared disease pathways [10]. Further factors possibly associated with the increased risk of T2D amongst patients with SCZ include ethnicity, obesity, a sedentary lifestyle, smoking, unhealthy dietary habits, hypertension, hyperlipidemia, social determinants of health, genetics, as well as long-term treatment with psychotropic drugs.

In order to investigate the epidemiological and genetic associations with cardiometabolic disease in patients with psychiatric disorders we established the GOMAP study (Genetic Overlap between Metabolic and Psychiatric disease), a new hospital-based collection of patients with psychiatric disease, with and without T2D. Commonly prescribed drugs include SGA, FGA, antidepressants, and mood stabilizers. These can be applied as monotherapy, 2-drug combination, or as 3- or more drug combinations in SCZ patients. A small number of studies have evaluated the potential diabetogenic effect of specific psychotropic drug classes.

Antidepressants and mood stabilizers have been individually associated with T2D risk. Associations between antidepressants [11–17], such as amitriptyline and mirtazapine, mood stabilizers [16, 18], such as lithium and valproate, modest weight gain and T2D have been reported, although the literature is still inconclusive [19]. A 9-year longitudinal study of the Data from an Epidemiological Study on the Insulin Resistance Syndrome (D.E.S.I.R. cohort) reported that the association between antidepressants use and diabetes might not be causal [20]. However, a recent meta-analysis indicated that antidepressants increase the likelihood of new-onset T2D [21]. A further cohort study conducted between 2005 and 2015 including 2579 SCZ patients revealed an independent association between antidepressants and risk of T2D for SCZ patients [9]. Furthermore, mood stabilizers have been found to be associated with an elevated risk for the development of insulin resistance in patients with epilepsy [22] or depression [23], whereas no association was found in patients with severe mental illness, with the exception of lithium [17]. Two studies have evaluated the effects of co-prescribed medications on T2D risk and both focused on youths exposed to limited medication regiments [24–26]. One of these studies [25] found a higher risk of T2D in patients receiving SGA together with antidepressants compared with patients receiving SGA only or antidepressants only.

A better understanding of the underlying mechanism linking T2D and psychiatric disorders can provide evidence and help shape strategies for the prevention of T2D in this group of patients. Here, we have studied risk factors linking pharmacological treatment and the risk of T2D in patients with SCZ.

## Methods

### Study population

**Fig. 1:**
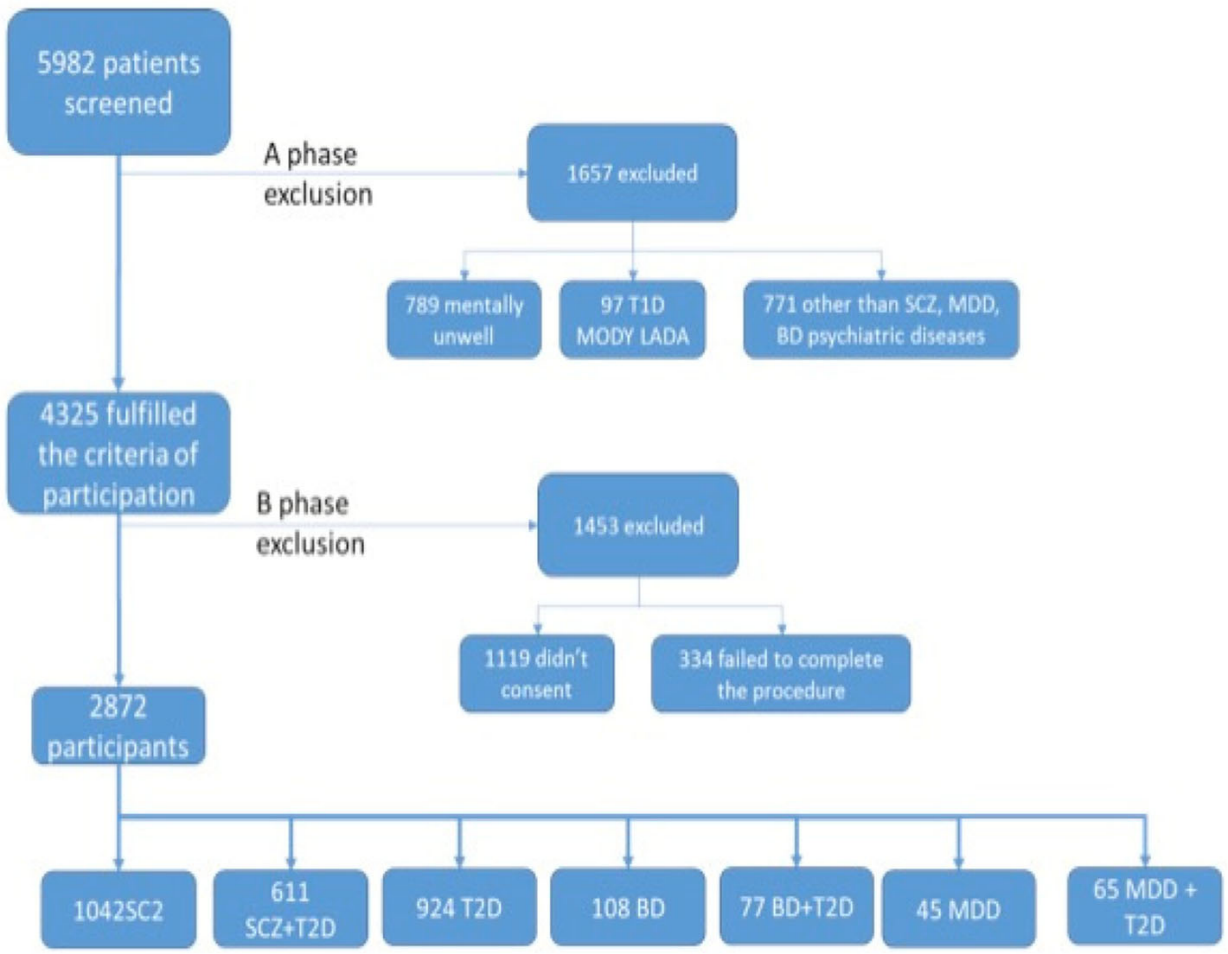
Study participant flow diagram. SCZ: schizophrenia, BD: bipolar disorder, MDD: major depressive disorder, T2D: type 2 diabetes

GOMAP is a nested case-control study. Participants were recruited from two hospitals in Athens, Greece (Dromokaiteio Psychiatric Hospital and Dafni Psychiatric Hospital). Briefly, in the work presented here, we randomly selected and screened 5985 patients. We excluded in total 4332 patients (Fig. 1) based on the following exclusion criteria: 1) age < 18 years, 2) presence of a psychiatric disorder other than SCZ, 3) presence of type 1 diabetes, latent autoimmune diabetes of adults, or gestational diabetes, 4) inability to provide informed consent due to mental state following consultation with the treating psychiatrist, 5) pregnancy or breastfeeding, and 6) non-Greek ancestry.

Ethical approval was obtained from the regulatory bodies of the participating hospitals. All study participants gave written informed consent following a detailed explanation of the study. This work has been carried out in accordance with the latest version of the Declaration of Helsinki [27].

### Medication data collection

The medication data sources used in the study include medical records from the hospital where the participant enrollment took place, as well as the database of the electronic prescription platform of the National Organization for Health Care Services Provision for the medication history. When gaps were detected in terms of treatment information accuracy, or in treatment compliance certainty, candidates were excluded. After careful quality checks of the medication data, the participants selected for inclusion had received one of the following four non-overlapping treatment categories consistently for a duration of at least six months: 1) monotherapy with FGA antipsychotics, 2) monotherapy with SGA antipsychotics, 3) combined therapy of two psychiatric medications, one of which antipsychotic and the second other class antipsychotic, antidepressant or mood stabilizer, and 4) triple or more psychiatric medications, one of which antipsychotic the other as in group before. Individuals switched to another treatment category were excluded from the study, however candidates switched to another regimen of the same category or additionally receiving another regimen of same category remained in the study. Recurrence of schizophrenia during this six months period was handled with application of parenteral aloperidin and benzodiazepine for no longer that 3 days, according to hospital’s protocol, a procedure that is not associated with T2D risk and was homogenously applied to all patients requiring it.

### Study procedure

The fieldwork was carried out between January 2012 and May 2014 and included an interview, a questionnaire concerning lifestyle habits, detailed medical history, treatment information, anthropometric measurements as well as a venous blood draw. Weight, height, waist and hip circumference were recorded for all individuals, and BMI and waist-to-hip ratio were calculated. Furthermore, syndrome profiles of the SCZ subjects were evaluated by performing the 30-item rating the Positive and Negative Syndrome Scale PANSS questionnaire, as adapted and validated for the Greek population [28].

Patients had received a diagnosis of SCZ, based on the structured clinical interview of the Diagnostic and Statistical Manual of Mental Disorders, (DSM-IV-TR) [29]. The diagnostic assessment of T2D was determined as defined by the American Diabetes Association [30]. Further, all individuals were asked to participate in an extensive screening of metabolic parameters.

### Statistical analysis

Comparisons between categorical and continuous variables were performed through a t-test. Nonparametric Mann-Whitney and Kruskal-Wallis tests were used due to the skewed distributions of some variables. Differences in proportions for outcome variables between groups were compared using Pearson’s chi-squared or Fisher’s exact test where appropriate. Multivariable logistic analyses were performed to examine associations between clinical important variables (sex, duration of SCZ, number of hospitalizations, psychiatric medication) and T2D status. Age and BMI were systematically introduced in the logistic model (adjusted results). The Benjamini and Hochberg procedure was used to correct for multiple testing [31]. Nominal statistical significance was set at *p* < 0.05. The analyses were performed using R (R version 3.2.2; The R Foundation for Statistical Computing, Vienna, Austria).

### Genotype analysis

A total of 1519 DNA samples from the GOMAP cohort (977 with SCZ, 542 with SCZ and T2D) were genotyped on the Illumina HumanCoreExome (Illumina, San Diego) chip. Following standard quality control procedures [32], we retained 1429 samples genotyped at 524271 markers. We imputed GOMAP together with two in-house Greek sample collections, ARGO and TEENAGE, using a merged reference panel of UK10K, 1000 Genomes Project and MANOLIS [33] (a Greek isolated population) haplotypes using the IMPUTEv2 software [34]. After filtering for Hardy-Weinberg equilibrium (*p* < 1 × 10^− 4^), IMPUTE v2 info score (info< 0.4) and minor allele frequency < 1%, there were 9,565,382 genetic variants in the dataset. We performed a GWAS on T2D status in the group of patients receiving a combination of three or more drugs (n_cases_ = 94, n_controls_ = 121), two of which had to be of the SGA or FGA class. We adjusted for the first 10 components derived from multidimensional scaling (MDS) of GOMAP together with TEENAGE [35] and ARGO. The power of this comparison is very low, but the data are available through the European Genome/Phenome Archive (EGA) for potential future meta-analyses.

## Results

The clinical and demographic characteristics of participants are displayed in Table 1, and treatment regimens are presented in Table 2. Of the 1653 participants with SCZ included in our analysis, 611 (36.96%) also had T2D. Our study achieved a collection of 3.2–5.9% of the entire Greek T2D and SCZ comorbid population, calculated according to the T2D general Greek population prevalence and to the European SCZ and T2D comorbidity prevalence, respectively. The whole sample included 672 women (40.7%) and 981 men (59.3%) with mean age 50.22 years (SD 14.03) and mean BMI 27.58 kg/m^2^ (SD 5.77).

**Table 1:** Characteristics of 1390 SCZ patients with T2D (*n* = 536) and without T2D (*n* = 854)

Characteristics	SCZ without T2D *n* = 854 (61.44%)	SCZ with T2D *n* = 536 (38.56%)	*Corrected* *p* value****
Mean (SD)*			
Age (years)	45.83 (12.98)	57.07 (12.25)	< 0.0001
SCZ duration (years)	18.57 (11.69)	26.30 (11.61)	< 0.0001
BMI (kg/m^2^)	25.97 (4.54)	30.56 (6.75)	< 0.0001
n (%)**			
Male	516 (60.42%)	310 (57.84%)	0.467
Female	338 (39.58%)	226 (42.16%)	
Number of subjects who reported outdoor physical activity in last week	16 (1.87%)	4 (0.75%)	0.172
Number of subjects who reported no outdoor physical activity in last week	838 (98.13%)	532 (99.25%)	
Median (IQR)***			
Number of Hospitalizations	4.00 (3.00–7.00)	3.00 (2.00–6.00)	< 0.0001
Positive Symptoms	31.00 (27.00–36.00)	32.00 (27.00–37.00)	0.532
Negative Symptoms	30.00 (26.50–34.00)	30.00 (25.00–34.00)	0.486
General Psychopathology Scale	55.00 (48.00–64.00)	54.00 (47.00–64.00)	0.179
PANSS total score	118.00 (105.00–130.50)	117.00 (103.00–131.00)	0.467

SD: standard deviation; IQR: interquartile range

^*^t-test, ^**^x^2^ test, ^***^Wilcoxon Rank Sum test, ****Corrected *p*-value using Benjamini and Hochberg method for the comparison between SCZ patients with and without T2D

**Table 2:** Psychiatric medication

	SCZ without T2D	SCZ with T2D
Monotherapy		
FGA	246 (17.7%)	127 (9.14%)
SGA	126 (9.06%)	89 (6.40%)
Combination Therapy		
2-Drug Combination (Antipsychotic + 1 more psychiatric medication)	361 (25.97%)	209 (15.04%)
≥3-Drug Combination (Antipsychotic + 2 or more psychiatric medications)	121 (8.71%)	111 (7.99%)

*FGA* first generation antipsychotics, *SGA* second generation antipsychotics

We considered four non-overlapping treatment categories: those receiving monotherapy with FGA antipsychotics (*n* = 373), those receiving monotherapy with SGA antipsychotics (*n* = 215), those under combined therapy of two psychiatric medications, one of which antipsychotic (*n* = 570), and those under triple or more psychiatric medications, one of which antipsychotic (*n* = 232). Consistent with the literature, our study confirms that obesity [36, 37] and increasing age [38, 39] represent independent risk factors for T2D in patients with SCZ (Additional file 1: Table S1). Adjusting for age and BMI (Table 3, column 1) the results indicated that sex (*p* = 0.063), SGA (*p* = 0.595), 2-drug combination (*p* = 0.937), duration of SCZ (*p* = 0.960), number of hospitalisations (*p* = 0.937) and outdoor physical activity in last week (*p* = 0.858), were not significantly associated with risk of T2D. However, the combined use of at least three psychotropic drugs was associated with higher risk of T2D (*p* = 0.035) compared with FGA monotherapy.

**Table 3:** Crude and adjusted odds ratios (OR) of T2D occurrence based on logistic regression modelling among patients with SCZ

Variables	Crude^a^		Adjusted^c^	
	OR (95%CI)	Corrected *p*-value	OR (95%CI)	*p*-value
Sex (Reference group = Female)	1.38 (1.06–1.81)	0.063	1.42 (1.08–1.85)^a^	0.0112
Psychiatric Medication (Reference group = FGA)				
SGA	1.27 (0.84–1.93)	0.595	1.27 (0.84–1.93)	0.259
2-Drug Combination	0.96 (0.69–1.33)	0.937	0.98 (0.71–1.35)	0.885
≥3-Drug Combination	1.75 (1.19–2.59)^b^	0.035	1.81 (1.22–2.69)^b^	0.003
Number of hospitalizations	1.00 (0.96–1.03)	0.937	0.99 (0.96–1.03)	0.749
Duration of SCZ	1.00 (0.98–1.02)	0.960	1.00 (0.98–1.02)	0.975
Outdoor Physical activity in last week: (reference group = No)	0.62 (0.16–2.38)	0.858	0.71 (0.19–2.66)	0.613

^a^adjusted for age and BMI

^b^Significant predictor of T2D among SCZ patients

^c^Adjusted for age, BMI, sex, duration of SCZ and number of hospitalizations

Furthermore, in multivariable logistic regression analysis that included adjustment for age, BMI, sex, duration of SCZ and number of hospitalizations, the combination use of at least three psychotropic medications [odds ratio (OR), 95% confidence intervals (CI)] [1.81 (1.22–2.69), *p* = 0.003] was significantly associated with higher risk of T2D (Table 3).

We examined the genetic relationship between psychotropic medication and T2D risk in the subset of individuals who were on a polypharmacy medication regimen in the GOMAP cohort. No significant findings were observed, likely due to small sample size and low power (Additional file 1: Figure S1 and S2).

## Discussion

We have established a novel case-control sample collection (GOMAP) with extensive phenotyping to examine clinical epidemiological factors that may increase the risk of T2D in patients with SCZ. We identify a previously unreported association between T2D and the concomitant prescription of three or more different psychotropic medication classes, including at least one antipsychotic, when compared to FGA monotherapy.

We do not identify SGA as a significant risk factor compared to FGA, despite high power to detect moderate effects (86% power to detect an OR of 2). Both FGA and SGA have been previously linked to an increased risk of T2D [40–43]. While some studies suggest that SGA use is associated with a higher risk of T2D development compared to FGA use [24, 44], others were inconclusive [42]. One possible explanation is that there is no difference in diabetogenic effect between SGA and FGA [45]. A recent meta-analysis [17] demonstrated that individual FGA and SGA drugs were associated with higher T2D risk, but did not find a significant difference in T2D risk between drugs of these classes. In addition, a systematic review of 22 randomized control trials detected no difference in glycemic effect of antipsychotics versus placebo [46].

It is also possible that we did not detect a difference in T2D risk between SGA and FGA use because of varying diabetogenic profiles among the individual drugs within these classes [47–49]. There is conflicting evidence for differential diabetogenic effects of antipsychotics of the same class [50]. For example [51], the SGA aripiprazole has been associated with decreased T2D risk in first-episode patients with SCZ, while olanzapine (SGA), low-potency FGAs and clozapine (SGA) have all been found to increase T2D risk [51]. Conversely, the SGA risperidone has been shown to confer a similar diabetogenic risk to FGAs [52]. A further study found no statistically significant differences in fasting glucose between patients receiving FGA and SGA when patients treated with clozapine and olanzapine were excluded [53]. Further, the inconsistency of these findings in the literature could be partly due to unmeasured confounding factors [54], such as genetic background. For example, the Australian National Survey of Psychosis has shown that antipsychotic treatment had no additional impact on T2D risk in those with a T2D family history, whereas antipsychotics increased T2D risk in those without a T2D family history [39].

A recent meta-analysis of studies on comorbid SCZ with T2D in China showed that duration of schizophrenia (> 10 years) is a significant risk factor for T2D development [55]. We observed similar statistically significant results, but no association when we dichotomized SCZ duration into less or more than 10 years (OR 1.46, 95% CI: 0.89–2.4). We do not identify association between PANSS psychopathology scores, examined separately for positive symptoms, negative symptoms and the total score with T2D risk. A single study has shown an inverse relationship between PANSS score and BMI in SCZ patients, but contains no information on T2D prevalence [56]. Moreover, we do not find an association between physical activity and T2D in SCZ; this may be due to the effect of hospitalization, which results in a largely uniform environment in terms of physical activity and nutrition.

Many combinations of different classes of medications were used for the management of SCZ in our sample; this does not allow us to perform robust sub-analysis to examine the diabetogenic effect of different classes of medications, because of the small number of patients in each category. Importantly, we identify a significant risk attributable to the potential diabetogenic effect of psychotropic combinations, which are frequently applied in SCZ treatment, though we attempted no such association analysis for antidepressants or mood stabilizers applied as monotherapy given our small sample size in these groups respectively. To date, most studies have not extensively considered other medication variables or their combinations. Similarly, a recent study [25] on youths identified a higher risk of T2D in patients receiving SGA together with antidepressants compared with patients receiving only SGA or only antidepressants. Moreover, no difference in T2D occurrence was detected between receiving stimulants concomitantly to SGA, as compared with SGA only. There is further acknowledgement that the increase in T2D risk in SGA-exposed youths remains small. Both studies emphasize the importance of including all psychotropic regimens received by SCZ patients when analyzing T2D risk to avoid missing synergy in this direction. Our study demonstrates the effect of combined psychiatric medication on T2D risk. Given the widespread use of combined categories of psychiatric medications, we suggest future studies should prospectively explore the effect of different treatment options on type 2 diabetes incidences, in order to formulate clinical guidelines.

A limitation of our study is that we only had access to retrospective medication information of up to six months prior to study commencement. This means that metabolic adverse effects of drugs beyond that period could not be accounted for and we cannot evaluate the effect of previous treatment regimens on T2D risk. Further we cannot make inferences about T2D causality, medication dose-effect, additional medication categories effect, differences in medication effects when patients are drug-naïve, or experience first SCZ episode; nor how effective, in terms of T2D prophylaxis, switching from one medication category to another might be. Additional factors that should be considered in a study of the metabolic effects of antipsychotic medications are duration of treatment [51, 57] and body weight before treatment initiation. Due to limited sample size it was not possible to study the effect of specific psychotropic medications, or various combinations thereof.

However, this study has examined a relatively large number of hospitalized participants, who have been carefully selected and ascertained for T2D and SCZ diagnoses based on established criteria rather than self-reporting. The homogeneity in the ethnicity of the studied population limits the potential for ancestry bias. Furthermore, our study is estimated to have collected 3.2–5.9% of the entire Greek T2D and SCZ comorbid population. We were further able to assess the effect of psychotropic medication in the context of SCZ alone, rather than having to account for several different psychiatric diagnoses [58]. Adherence to treatment was strictly inspected.

While the genetic analysis did not yield any robust signals, it might prove useful in downstream meta-analyses. The genotype data used here have been made available through the European Genome/Phenome Archive.

## Conclusions

Our findings indicate that a triple-drug combination therapy for patients with SCZ is associated with increased risk of T2D compared with treatment involving FGA. Future studies should explore the research hypothesis that maximization of monotherapy doses or switch to a different antipsychotic class may be associated with decreased diabetogenic effect as compared with the addition of multiple antipsychotic medications.

## Additional file

Additional file 1: Combination therapy as a potential risk factor for the development of type 2 diabetes in patients with schizophrenia: the GOMAP study. Description of survey flow. Diagnostic assessment of T2D and psychiatric disease. Physical assessments. **Table S1.** T2D occurrence based on simple logistic regression modelling among patients with SCZ. **Figure S1.** Manhattan plot of association results from a GWAS on T2D in individuals receiving a combination of three or more psychotropic drugs, including one FGA and one SGA. For each of the 9,565,382 analysed variants the –log10 of the *p*-value is plotted against its chromosomal position. Blue and red lines indicate suggestive (*p* < 5*10^− 6^) and genome-wide (*p* < 5*10^− 8^) significance. **Figure S2.** Quantile-quantile plot of association results from a GWAS on T2D in individuals receiving a combination of three or more psychotropic drugs, including one FGA and one SGA. For each SNP the –log10 of the p-value is plotted against its expected value under the null distribution. (DOCX 219 kb)

## Abbreviations

BMI: Body mass index
CI: Confidence intervals
D.E.S.I.R.: Epidemiological Study on the Insulin Resistance Syndrome
DSM-IV-TR: Diagnostic and Statistical Manual of Mental Disorders
EGA: European Genome/Phenome Archive
FGA: First-generation antipsychotics
GOMAP: Genetic Overlap between Metabolic and Psychiatric disease
MDS: Multidimensional scaling
OR: Odds ratios
PANSS: Positive and Negative Syndrome Scale
SCZ: Schizophrenia
SD: Standard deviation
SGA: Second-generation antipsychotics
T2D: Type 2 Diabetes
