# Supplementary material for: Combination therapy as a potential risk factor for the development of type 2 diabetes in patients with schizophrenia: the GOMAP study
Source: BMC Psychiatry. 2018 Aug 2;18:249. doi: 10.1186/s12888-018-1826-4 (PMC6090901; doi:10.1186/s12888-018-1826-4)
Supplement: Supplementary file 1 — Combination therapy as a potential risk factor for the development of type 2 diabetes in patients with schizophrenia: the GOMAP study. Description of survey flow. Diagnostic assessment of T2D and psychiatric disease. Physical assessments. Table S1. T2D occurrence based on simple logistic regression modelling among patients with SCZ. Figure S1. Manhattan plot of association results from a GWAS on T2D in individuals receiving a combination of three or more psychotropic drugs, including one FGA and one SGA. For each of the 9,565,382 analysed variants the –log10 of the p-value is plotted against its chromosomal position. Blue and red lines indicate suggestive (p < 5*10− 6) and genome-wide (p < 5*10− 8) significance. Figure S2. Quantile-quantile plot of association results from a GWAS on T2D in individuals receiving a combination of three or more psychotropic drugs, including one FGA and one SGA. For each SNP the –log10 of the p-value is plotted against its expected value under the null distribution. (DOCX 219 kb) [file 12888_2018_1826_MOESM1_ESM.docx]

Additional file 1

Study population-Description of survey flow

The GOMAP study is a case-control study on unrelated Greek nationals aiming to estimate clinical and genetic correlations between type 2 diabetes mellitus (T2D) and psychiatric diseases. Volunteers were recruited randomly and out of 5985 patients screened for the purposes of the study, 2875 eventually participated in the study. All patients with a diagnosis of schizophrenia (SZC), bipolar disorder (BD) or major depressive disorder (MDD) based on the *Diagnostic and Statistical Manual of Mental Disorders,* fourth edition (DSM-IV) and individuals with T2D, without psychiatric comorbidity were asked to participate in an extensive screening of metabolic parameters. 1657 were excluded for not meeting the criteria of the study (age<18, other psychiatric diseases than SCZ, MDD and BD, type 1 diabetes, maturity onset diabetes of the youth (MODY), latent autoimmune diabetes of adults (LADA), mentally unwell to provide consent judged by the case physician each time). 1119 didn’t consent and 334 failed to compete the procedures of the survey (Additional file 1: Figure S1). Participants were recruited from four hospitals in Athens, Greece. Psychiatric disease patients were inpatients recruited from Dromokaiteio Psychiatric Hospital and Dafni Psychiatric Hospital, whereas T2D participants were collected from diabetes outpatient clinics at Hippokrateio General Hospital and Laiko General Hospital. The latter set of T2D patients have not been used in the analyses reported here. Ethical permission was obtained from the appropriate regulatory bodies of the participating hospitals and all volunteers gave written informed consent after an in-depth explanation of the aims of the GOMAP study. This work has been carried out in accordance with the latest version of the Declaration of Helsinki[[27](#_ENREF_27)].

The study sample of 2871 individuals aged over 18 was divided into three subgroups; individuals with T2D and psychiatric disease (hospitalized), individuals with psychiatric disease without T2D (hospitalized), and individuals without psychiatric disease and with T2D (outpatients) and further segmented into 7 categories; T2D, T2D with SCZ, SCZ, T2D with BD, BD, T2D with MDD, MDD.

This specific study is based on a subsample of the GOMAP study and specifically on the participants with SCZ with and without T2D, including a total of 672 females (40.7%) and 981 males (59.3%), with a mean age of 50.22 years (S.D. = 14.03) and a mean BMI of 27.58 kg/m^2^ (S.D. 5.77). Furthermore, the mean number of hospitalizations for the whole sample size was 4.712 (S.D. 3.81), with a mean duration of SCZ diagnosis of 21.52 years (S.D. 12.43). Given the 11.97% and 0.8% prevalence of T2D[[4](#_ENREF_4), [7](#_ENREF_7)] and SCZ[[58](#_ENREF_58)] in Greece, respectively, the European calculated prevalence of T2D and SCZ comorbidity, which is evaluated up to 22% and an estimated Greek population size of 10,816,286 according to Hellenic Statistical Authority[7], we expect there to be 86,531 patients with SCZ in the general Greek population and among them 10,400 - 19,000 with T2D comorbidity, according to Greek general population T2D prevalence and European SCZ+T2D comorbidity prevalence rates.

The fieldwork was carried out between January 2012 and May 2014, which included volunteer recruitment, interview and completion of a detailed questionnaire followed by a single blood draw.

Diagnostic assessment of T2D and psychiatric disease

The diagnostic assessment of T2D was determined as defined by the American Diabetes Association [[30](#_ENREF_30)]: Fasting plasma glucose (FPG) ≥ 7.0 mmol/l (126 mg/dl) or, 75 g oral glucose tolerance test (OGTT) with FPG ≥ 7.0 mmol/l (126 mg/dl) and/or 2 hour plasma glucose ≥ 11.1 mmol/l (200 mg/dl) on two different days or, random plasma glucose ≥ 11.1 mmol/l (200 mg/dl) in the presence of classical diabetes symptoms of diabetes mellitus. In the case where a random plasma glucose level ≥ 5.6 mmol/l (≥ 100 mg/dl) and < 11.1 mmol/l (< 200 mg/dl) was detected, a FPG was measured, or an OGTT was performed.

Subjects with psychiatric diagnoses eligible for our study were screened in the appropriate departments where diagnosis was confirmed. The psychiatric disease participants were hospitalized and had undergone psychiatric interviews from consultant psychiatrists. Psychiatric diagnosis was set according to the Diagnostic and Statistical Manual of Mental Disorders DSM-IV criteria, which are applied in the specific hospitals.

Syndrome profiles of the SCZ subjects were evaluated by performing the Greek version of 30-item rating Positive and Negative Syndrome Scale (PANSS), as adapted and validated for the Greek population by Lykouras et al [[28](#_ENREF_28)].

Physical assessments

Weight and height were recorded for all individuals, whereas body mass index (BMI) was calculated. Subjects were weighed, with minimal necessary clothing, with weight balance scales and height was measured with a stadiometer. Further factors associated with T2D such physical activity and pharmacotherapies were also recorded.

**Additional file 1: Table S1. T2D occurrence based on simple logistic regression modelling among patients with SCZ**

| **Variables** |  | |
| --- | --- | --- |
|  | **OR (95%CI)** | ***Corrected***  ***p*-value** |
| **Sex**  **(Reference group=Female)** | 0.9 (0.72-1.12) | 0.002 |
| **BMI** | 1.18 (1.15-1.21) ^a^ | < 0.001 |
| **Age** | 1.07 (1.06-1.08) ^a^ | < 0.001 |
| **Psychiatric Medication**  **(Reference group=FGA)** |  |  |
| **SGA** | 1.37 (0.97-1.93) | 0.381 |
| **2-Drug Combination** | 1.12 (0.85-1.47) | 0.412 |
| **≥3-Drug Combination** | 1.78 (1.27-2.48) ^a^ | 0.113 |
| **Number of hospitalizations** | 1.05 (1.02-1.08) ^a^ | 0.002 |
| **Duration of SCZ** | 1.06 (1.05-1.07) ^a^ | < 0.001 |
| **Outdoor Physical activity in last week:**  **(reference group=No)** | 0.39 (0.13-1.18) | 0.125 |

**
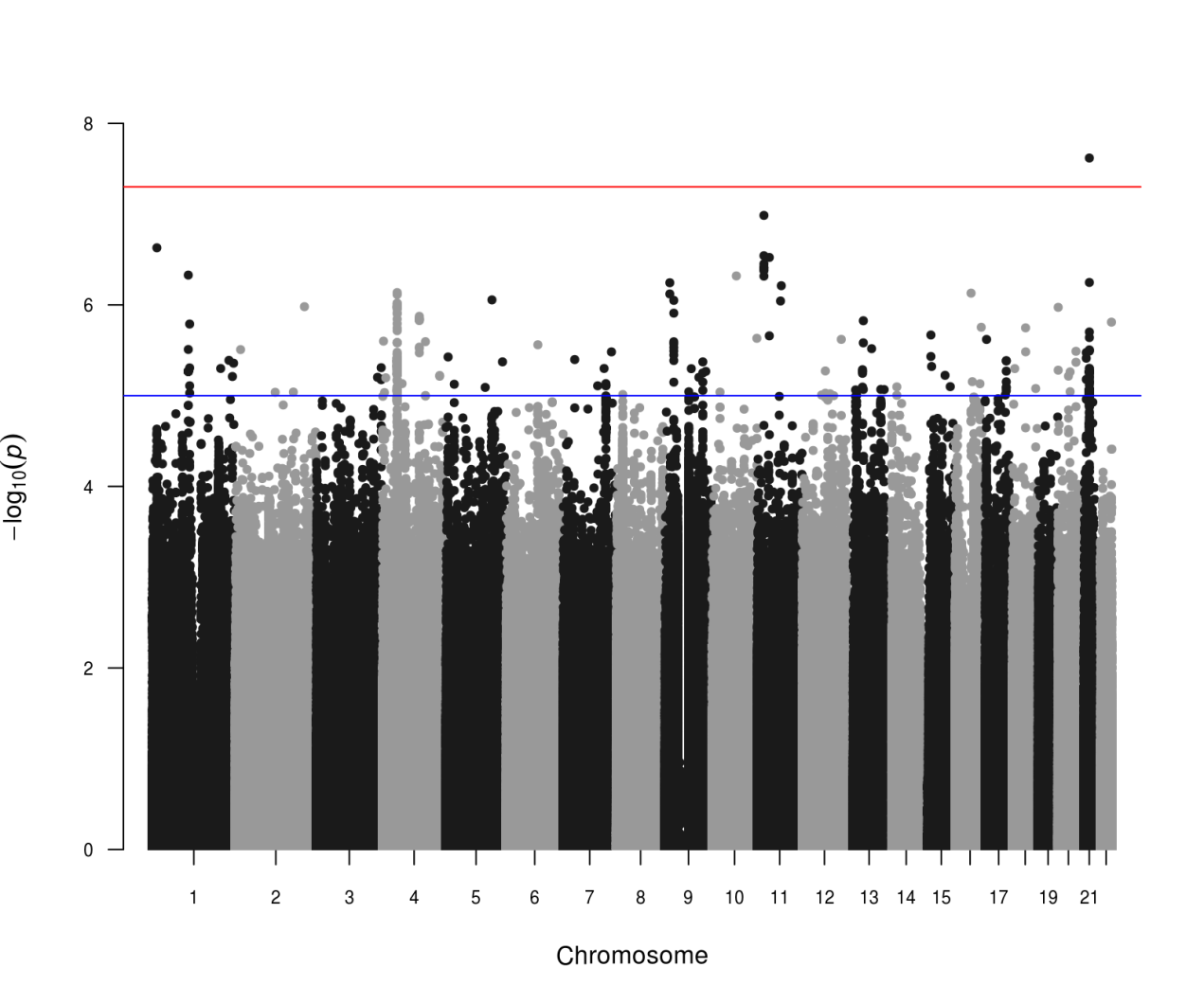
**

**Additional file 1: Figure S1. Manhattan plot of association results from a GWAS on T2D in individuals receiving a combination of three or more psychotropic drugs, including one FGA and one SGA. For each of the 9565382 analysed variants the –log10 of the p-value is plotted against its chromosomal position. Blue and red lines indicate suggestive (p<5*10^-6^) and genome-wide (p<5*10^-8^) significance.**

**
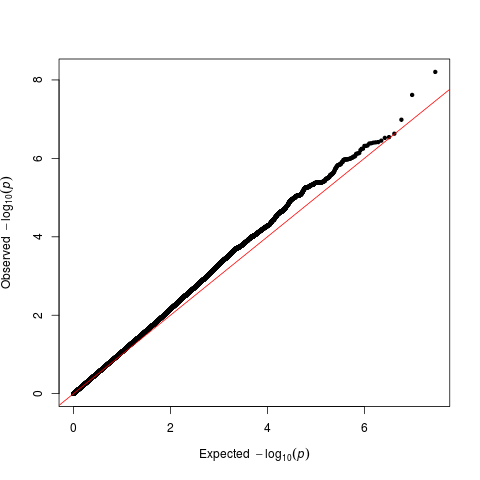
**

λ=1.101

**Additional file 1: Figure S2. Quantile-quantile plot of association results from a GWAS on T2D in individuals receiving a combination of three or more psychotropic drugs, including one FGA and one SGA. For each SNP the –log10 of the p-value is plotted against its expected value under the null distribution.**
